# Healthcare provider recommendations to improve post‐violence care HIV post‐exposure prophylaxis access and adherence in Mozambique

**DOI:** 10.1002/jia2.26452

**Published:** 2025-06-26

**Authors:** Meghan Duffy, Etevaldo M. F. Xavier, Anabela de Almeida, Della Correia, Maria Nhavane dos Prazeres, Jacinto Adriano, Bainabo Parruque, Maria Olga Bule, Langan Denhard, Maura Almeida, Ana Baptista, Raquel Cossa de Pinho

**Affiliations:** ^1^ Division of Global HIV & TB U.S. Centers for Disease Control and Prevention (CDC) Maputo Mozambique; ^2^ Friends in Global Health Maputo Mozambique; ^3^ Jhpiego Maputo Mozambique; ^4^ Ministry of Health Maputo Mozambique; ^5^ U.S. Centers for Disease Control and Prevention (CDC) Atlanta Georgia USA

**Keywords:** post‐exposure prophylaxis, post‐violence care, sexual violence, intimate‐partner violence, HIV, access

## Abstract

**Introduction:**

In Mozambique, post‐exposure prophylaxis (PEP) to prevent HIV is offered as part of the essential package of post‐violence care services at 1450 health facilities. However, HIV PEP access and adherence continue to be a challenge. Healthcare providers were interviewed to identify and synthesize their recommendations for improving PEP access and adherence.

**Methods:**

We conducted semi‐structured, in‐depth interviews with 20 adolescent and adult healthcare providers (3 men and 17 women) who had a range of 2−15 years of experience from 20 health facilities across seven provinces during March–August 2023. Data were analysed using inductive and theoretical thematic analysis. We analysed how frequently health providers mentioned specific recommendations.

**Results:**

Regarding PEP access, healthcare providers recommended community education as the most effective strategy (10 mentions). In particular, providers cited the importance of *palestras* [community health talks]. Providers also commonly highlighted the need to have PEP kits prepared (7 mentions) and PEP readily available at health facilities (6 mentions). Regarding PEP adherence, providers recommended client counselling/education (13 mentions) to ensure clients understand the importance of taking PEP, how to properly take PEP and the potential side effects, which can often deter clients from adhering. Additionally, providers highlighted *chamadas preventivas* [follow‐up telephone calls] within 2 weeks or so after the initial visit (9 mentions) as the best means to ensure clients complete the full, 28‐day regimen and return for retesting after 3 months. Healthcare providers explained that follow‐up telephone calls, despite the client living far from the health facility, can create a bond that supports clients. Providers recommended the institutionalization of follow‐up telephone calls for consistent implementation in all healthcare facilities that offer PEP.

**Conclusions:**

Interviewed healthcare providers offered valuable insights and recommendations to improve PEP access and adherence, which could be considered for implementation in Mozambique and other sub‐Saharan African countries.

## 1 INTRODUCTION

Worldwide, 31% of adolescent girls and women aged 15–49 years have experienced violence at some point in their lives, leading to both short‐ and long‐term health consequences including HIV [1]. Women experiencing intimate partner violence (IPV) are 1.5 times more likely to acquire HIV compared with women who had not experienced IPV [2]. Mozambique has established and scaled up violence prevention and response within existing HIV clinical services over the past decade from an initial mere 56 public health facilities providing post‐violence care services in 2012 to 1450 public health facilities providing post‐violence care services in 2021—an overall 81% coverage of the total 1778 existing health facilities [3].

The essential package of post‐violence care services has been defined by the Mozambique Ministry of Health (MoH) and the President's Emergency Plan for AIDS Relief (PEPFAR) to include provision of clinical services, rapid HIV testing with referral to care and treatment as appropriate, sexually transmitted infection (STI) tests/treatment, emergency contraception, additional counselling and referrals as needed (police, legal, psychosocial support, etc.) and HIV post‐exposure prophylaxis (PEP).

HIV PEP is an effective HIV‐prevention method that consists of administering antiretrovirals to HIV‐seronegative individuals exposed to a potential risk of HIV acquisition for 28 days, starting within 72 hours of exposure [4, 5, 6, 7, 8, 9]. In 2005, the MoH published the first PEP guidelines, initially for healthcare providers exposed to HIV at work [10]. In 2011, the national guidelines were revised to expand PEP provision to all health facilities that offered post‐violence care services. Since then, HIV PEP has been offered in Mozambique as part of the essential package of post‐violence care services [10].

Despite this progress, PEP access and adherence has been a challenge in Mozambique. A study in Mozambique's Zambezia Province demonstrated that almost 60% of survivors of sexual violence arrived more than 72 hours after exposure and were, thus, ineligible for PEP [11]. This trend is not unique to Mozambique. Results from across 15 PEPFAR countries in sub‐Saharan Africa (SSA) revealed that PEP coverage was 15% among older adolescents aged 15–19 years who experience sexual violence [12]. This statistic is particularly concerning because adolescent girls and women aged 15–24 years are at the highest risk for new HIV acquisition in SSA and at the highest risk for sexual violence [13, 14]. Additional studies have demonstrated poor HIV PEP adherence among a variety of populations [9, 15]. A systematic review and meta‐analysis of PEP adherence revealed that the proportion of people considered eligible for PEP who completed the full 28‐day course was 56.6%. Completion rates were lowest for survivors of sexual violence at 40.2% [16].

Considering these challenges, healthcare providers throughout Mozambique were interviewed to identify and synthesize their recommendations towards improving PEP access and adherence.

## 2 METHODS

### 2.1 Data collection

Semi‐structured in‐depth interviews (IDIs) with 20 health providers from 20 health facilities across seven provinces were conducted from March to August 2023. The individual interviews took place in a private room in the health facility so that interviewees could speak comfortably and in confidence. The interviews lasted approximately 30–45 minutes and were conducted in Portuguese by two, lead researchers on the team (ADA and MOB). The interviews followed a semi‐structured format based on the interview guide developed by the research team to better understand the strengths/weaknesses of post‐violence care in Mozambique as well as elicit recommendations to promote PEP access and adherence. The interview guide domains were as follows: socio‐demographic and professional data; role of the provider, provider responsibilities; strengths of post‐violence services; provider perspectives regarding strengths; weaknesses of post‐violence services; provider perspectives regarding weaknesses; violence prevention activities; positive provider abilities; recommendations to promote PEP access and adherence.

The interviews were conducted, audio‐recorded and transcribed by three members of the research team (ADA, MOB and MA). The transcriptions were then uploaded into MaxQDA for coding and analysis in Portuguese. Data on participants’ position, sex and age were collected, but participant name and contact information were not recorded. Interviews were stripped of all unintentional identifiers during transcription and interviewees provided code numbers and names.

### 2.2 Sampling

Purposive sampling using a theoretical sampling approach was employed. We sought “information‐rich cases” described by Patton as “those from which one can learn a great deal about issues of central importance to the purpose of the research” [17]. We interviewed one manager or healthcare provider (sometimes the same person served both roles) of adolescent and adult post‐violence services in 20 health facilities where we simultaneously conducted external quality assessments. External quality assessments were conducted in 50 health facilities that met 80% of the quality assessment criteria according to a structured quality assurance tool designed to assess post‐violence clinical services [18]. Availability of the research team and geographic representation were also taken into consideration when selecting the health facilities where interviews would be conducted. The manager/healthcare providers we interviewed were selected according to the inclusion criteria (provide violence‐related care for > 6 months, attended >1 violence course) and exclusion criteria (does not provide violence‐related care, provide violence‐related care for <6 months, attended < 1 violence course). Interviews were conducted until saturation was reached—that is: each additional interview was redundant, and there were diminishing returns on time spent conducting the interview. Saturation was reached after 20 interviews, and we did not conduct additional interviews during the external quality assessments.

A majority of the interviews were conducted in the southern region, in primary and urban health facilities. Over half the health facilities received over 100 cases of violence per year and half provided care to over 5000 clients in HIV care and treatment (Table 1).

**Table 1:** Characteristics of healthcare facilities included in the qualitative study of post‐exposure prophylaxis access and adherence in Mozambique, 2023

Healthcare facility characteristics	Number of facilities	Percent
Region		
South	13	65%
Centre	4	20%
North	3	15%
Centre for Integrated Attendance (CAI)	3	15%
Level of healthcare facility		
Primary	16	80%
Secondary	4	20%
Tertiary	0	0%
Urban or rural		
Urban	13	65%
Rural	7	35%
Cases of violence: 2022		
0−100	9	45%
101−300	8	40%
301+	3	15%
Patients on HIV treatment: 2022		
0−3000	4	20%
3001−5000	6	30%
5001−10,000	7	35%
10,001−16,000	3	15%

*Note*: A wide variety of healthcare providers with numerous years of experience and both classroom and on‐the‐job trainings were interviewed (Table 2)—ensuring we obtained “information‐rich cases” [17].

**Table 2:** Characteristics of healthcare providers interviewed in the qualitative study of post‐exposure prophylaxis access and adherence in Mozambique, 2023

Healthcare provider characteristics	Number of facilities	Percent
Professional category		
Doctor	2	10%
Nurse—Superior level	4	20%
Nurse—Mid level	4	20%
Psychologist	3	15%
Superior technician	3	15%
Medical technician	3	15%
Sex		
Male	3	15%
Female	17	85%
Years in category		
1−4	9	45%
5−9	8	40%
10+	3	15%
Years worked in health facility		
1−4	12	60%
5−9	7	35%
10+	1	5%
Number of classroom trainings		
1−4	13	65%
5−9	4	20%
10+	3	15%
Number of on‐the job trainings		
0−4	9	45%
5−9	8	40%
10+	3	15%
Post‐violence care role: service provision/management/both		
Both	15	75%
Service provision only	5	25%
Number of post‐violence care clients per month (avg)		
4−9	6	30%
10−15	6	30%
16−30	6	30%
30+	4	20%
Number of providers trained to offer post‐violence care		
4−9	8	40%
10−14	7	35%
15−30	5	25%
Year health facility began to offer post‐violence care		
2010−2015	8	40%
2015−2020	10	50%
N/A	2	10%

### 2.3 Data management

Consent forms were secured in a locked cabinet. Electronic data including audio files and transcriptions were stored on a password‐protected network. Electronic data were backed up weekly. Access to all information was limited to study staff.

### 2.4 Data analysis

Inductive and theoretical thematic analysis was used to study the data. Thematic analysis is a method for identifying, analysing and reporting patterns (themes) within data. It assists to organize and describe the data set [19]. The themes were both driven by the data (inductive) and the analyst (theoretical). The data analysis involved several steps. After an initial round of data collection (roughly 10 interviews), the research team of three (MD, ADA and MOB) coded two interviews together in order to identify emerging themes and develop the codebook using a standard iterative process. The codes were as follows: strengths, weaknesses, provider role, provider responsibilities, violence prevention activities, positive provider abilities, PEP access and adherence recommendations. The emerging themes were used to inform probes in subsequent interviews. Once data collection was complete, the team divided the remaining transcripts. After each team member coded her respective interviews, we reviewed and discussed coding approaches as well as the codebook.

After coming to a consensus on each coded interview, codes and data sets were merged. The codebook, merged/refined codes and coded interviews were circulated for final review and consensus among the group. Upon reaching a consensus, tables were created to consolidate the number of times providers mentioned specific recommendations regarding PEP access and adherence. We did not include multiple mentions by the same provider. For each of the top four recommendations mentioned, significant statements, previously identified through memos by researchers, were pulled from the transcripts. The significant statements highlight key aspects of the recommendations and further facilitate understanding. Tables and significant statements were then translated into English by one member of the research team (MD) and reviewed for accuracy and concordance by two additional members of the research team (ADA and MOB).

### 2.5 Ethics statement

All research participants provided written consent, and no reimbursements were offered for participation. The data are covered by the Violence Umbrella Protocol (Project ID #: 0900f3eb81ac9ed9), reviewed by the Mozambique MoH Institutional Review Board and U.S. CDC, deemed not research, and conducted consistent with applicable U.S. federal law and U.S. CDC policy (45 C.F.R. part 46.102(l)(2), 21 C.F.R. part 56; 42 U.S.C. Sect. 241(d); 5 U.S.C. Sect. 552a; 44 U.S.C. Sect. 3501 et seq).

## 3 RESULTS

### 3.1 PEP access

Regarding PEP access, healthcare providers mentioned community education as the most effective strategy (10 mentions), followed by the preparation of complete PEP kits (7 mentions) and available medication (6 mentions).

Health providers cited the importance of group *palestras* [health talks]. *Palestras* are normally given in the markets or other open spaces where large groups of community members gather. They generally last 15−20 minutes and cover a specific health topic. One provider asserted:

“Best approach is… the *palestras*. Because if we don't do *palestras*, they have no way to know…that information that the health facility provides services they will not know. So, we have to get out, get down from the place of comfort to the field. Yes.”

A second provider asserted:

“Then the recommendation is for the community, that has to know the key messages, that immediately after sexual violence he/she has to go to a health facility. And arrive as early as possible to be able to receive the package.”

The assertions that being out of their “place of comfort” and in the community to disseminate information to the people who need it the most was often frequently followed by the recommendation to ensure that the PEP kit is prepared (7 mentions) and medication is readily available and accessible once the patient arrives at the health facility (6 mentions). A complete PEP kit in Mozambique includes PEP (child and adult dosages), an HIV test, tests/prophylaxes for the most common STIs (gonorrhoea, syphilis, trichomoniasis and chlamydia), hepatitis B test/vaccine, pregnancy test, emergency contraception and paracetamol [10].

One provider recommended:

“Have the kits up to date, with complete medicines, with all of the alternative lines [of medication]. We know how to give priority to the victims so as not to waste time—or we will miss the opportunity. Because if she arrives for example at night and the person says: come back tomorrow, we don't know what time the violence was, and we risk her coming back tomorrow after 72 hours.”

This recommendation was echoed by several other providers who acknowledged that if PEP is not available, the opportunity to administer PEP may be missed.

### 3.2 PEP adherence

Regarding adherence, providers recommended client counselling or education at the facility as the most effective strategy (13 mentions), followed by follow‐up telephone calls (9 mentions) and general follow‐up (5 mentions). They emphasized the need to ensure clients completely understand the importance of taking the medication, how to properly take the medication and the potential side effects.

One provider summarized:

“Counsel and encourage. Yes. For her to feel confident… to value what she is taking away, knowing that this is going to transform my life, in a better way. So, it is on the basis of this counseling that she will be able to see that in fact my life is here. We have to be aware, we have to have empathy, let her know that I'm here to support. She walks away with that image that it's in that hospital, it's that person that I put my trust in.”

Another provider emphasized that efforts to counsel have not been achieved if the patient does not understand the importance of taking the medications (despite side effects) and returning for follow‐up visits:

“Counseling, because the person can take it and throw it away. Can take the medicine, get home, take it, have… reactions, some anomalies and vomiting, intestinal pains, do not know what. And she stops. That is why the majority of victims do not return after—efforts to counsel are weak. It is counseling. The person counsels, but does not achieve the goal of counseling, which is to make the victim return. Counseling is not just talking. It's not just saying haa you have to return—you have to take the last test. No—counseling is convincing the person of the importance of returning.”

A third provider delved into great detail regarding the need to ensure that patients understand the potential negative side effects in order to achieve adherence:

“To ensure good adherence to these medications we have to explain very well what are the side effects of the medications. Because if we don't explain, she takes it, we know the antiretrovirals have… sometimes cause nausea, headache, then vomiting, diarrhea, abdominal disorders. If we don't explain this, when she goes home, starts taking the medications, has these symptoms—stops. So, we have to explain very well the side effects of the drugs, and also explain the importance of taking the drugs.”

Additionally, providers highlighted *chamadas preventivas* [follow‐up telephone calls] within 2 weeks or so after the initial visit (9 mentions) as the best means to ensure clients complete the full regimen and retest after 3 months. Health providers explained that preventive telephone calls, despite the client living far from the health facility, can create a bond that supports clients.

One provider stated:

“Doing a follow‐up call, maybe after 15 days, would help, right? Understanding from a distance. Haa—okay, you started, I don't know what—how do you feel, did you have any adverse effects? And maybe in these 15 days we could, right, have feedback, that okay, I'm tired I don't want to continue—we will reinforce it, right? The need to continue and finish the prophylaxis.”

A second provider was adamant that it is possible to create a bond with the patient even through follow‐up calls:

“Yes, that's exactly what I said—talking with the victim, creating that bond, him feeling comfortable, he feels welcome. I believe that exchanging numbers, talking, because the cases that we have are few, it's doable. It is possible for us to communicate: Where are you? How are you? Your husband? These days, what is it like? It's possible. It's the only thing we can do.”

Health providers recommended the institutionalization of *chamadas preventivas* for consistent implementation in all health facilities. The following quote is an example of this recommendation:

“Well, I think the Ministry should design a scheme just like the HIV program because ART comes from the HIV program. There they have calls to see if the person is taking the medication well—so, these are strategies that the health facility implements that after 7 days you have to call…reminder calls for the person to adhere. We had people returning because many times these people… then they feel more welcome.”

## 4 DISCUSSION

From IDIs with 20 healthcare providers in Mozambique, we obtained insightful information about improving PEP access and adherence. Interviewed providers recommended community education to improve PEP access. Providers repeatedly expressed that people need to know that HIV PEP is available and that it must be taken within 72 hours of a violence‐related exposure. Interviewed providers were confident that with active community engagement via *palestras*, information about PEP availability can be appropriately disseminated, and survivors of violence can arrive at health facilities in time to receive essential services. Providers detailed several examples of increases in community members seeking PEP at health facilities after simple, local and low‐cost community outreach activities had been conducted in nearby communities. This finding is consistent with a study in Zambezia Province that advocated for community‐wide and targeted educational initiatives to increase the uptake of services within the 72‐hour window [11]. Other studies conducted in nearby SSA countries also recommend the dissemination of information in the community as a means to address barriers to post‐violence service uptake [12, 20, 21, 22]. Furthermore, the recommendation aligns with global guidelines on improved access to HIV PEP through community facilities and services [9]. This community education can be conducted by community partners and local leaders with minimal costs in order to ensure the timely arrival of survivors. A promising intervention out of Mombasa, Kenya used paralegals to serve as focal points for community engagement—conducting community dialogues to educate the community, identify survivors and engage them to seek post‐violence services [23].

Client counselling/education on the importance of completing the PEP regimen was strongly recommended by providers to improve PEP adherence. Providers explained that clients are likely to stop taking PEP if they do not understand the importance of completing the regimen or if they do not understand the side effects. This observation is supported by the literature. An article on patient adherence explains that adherence barriers include insufficient explanations of adverse effects and lack of communication regarding lifestyle and economic conditions [24, 25]. Global guidelines also recommend enhanced adherence counselling for individuals initiating HIV PEP due to several studies that demonstrated the effectiveness [7, 9, 26, 27].

Another recommendation mentioned by providers to ensure PEP adherence was *chamadas preventivas*, within 2 weeks or so after the initial visit, to check in on clients and inquire about side effects, motivate the client to continue treatment and remind the client to return for testing after he or she finishes the full course of medication. Several providers mentioned the need for the MoH *Programa de Violência‐Baseado no Gênero* [Gender‐Based Violence Program] to institutionalize this strategy as has been done by the MoH HIV Program [28]. Various studies demonstrate that reminders improve patient adherence and that regular telephone reminders, emphasizing the importance of treatment adherence, are effective in enhancing adherence [29, 30, 31, 32]. Reminders are also one of the least costly interventions [25].

Follow‐up telephone calls are a recommended practice that the *Programa de Violência‐Baseado no Gênero* has begun to adopt [33]. These follow‐up calls currently occur at a handful of health facilities but are not widely used. The programme plans to make the follow‐up calls a routine practice at all healthcare facilities that offer PEP.

In fact, the MoH plans to implement all of the provider recommendations during 2025 [33]. For example, a PEP community education campaign called *Cada Hora Conta* [Every Hour Counts] revised Standard Operating Procedures to address PEP kit preparation/PEP availability and additional trainings on PEP adherence counselling are currently being developed by a national technical working group for subsequent dissemination and implementation thereafter.

Considering that PEP access and adherence are also low across many sub‐Saharan African countries with similar contexts [9, 12, 15], confronting similar challenges such as staffing and resource shortages, we posit that the research findings might also be useful to other SSA countries.

Furthermore, although the focus of this study was on PEP access and adherence in instances of HIV exposure due to violence, provider recommendations might also serve to improve access and adherence in instances of HIV exposure via other routes—occupational, sharing needles and so on. In 2020, Mozambique MoH PEP Guidelines were updated to include HIV PEP administration in instances of all potential HIV exposures. However, HIV PEP is still primarily administered in instances of HIV exposure due to violence. Discussions regarding the utility of the study's findings in instances of HIV exposure via other routes are underway at the Mozambique MoH [10]. Furthermore, discussions within the MoH regarding the adoption of global guidance for increasing PEP access through community distribution and task‐shifting within the Mozambique context have begun [9].

### 4.1 Limitations

A limitation of the IDIs is that the providers interviewed were often the most experienced post‐violence healthcare providers present—trained and well‐versed in the provision of post‐violence services. Therefore, the providers were often champions for the improvement of post‐violence services and their opinions and knowledge of post‐violence services are most likely not representative of other providers. A second limitation of the study is the potential for social‐desirability bias due to the nature of one‐on‐one interviews. The research team encouraged honesty for programme improvement purposes and ensured participants that their responses would not be linked to their names or any other identifiable information.

## 5 CONCLUSIONS

Interviewed healthcare providers offered valuable insights and recommendations to improve PEP access and adherence, which will be implemented in Mozambique and could be considered for implementation in other sub‐Saharan African countries.

## COMPETING INTERESTS

The authors declared no potential conflicts of interest with respect to the research, authorship and/or publication of this article.

## AUTHORS’ CONTRIBUTIONS

MD, ADA, EMFX and RCDP developed the study design. MD, ADA and MOB led the data collection. MD, ADA, MOB and MA led the data analysis and interpretation. MD led the manuscript development and revisions. All authors significantly contributed to the data collection, analysis and manuscript development. The manuscript underwent a review by all authors, and each one approved the final version.

## FUNDING

This research has been supported by PEPFAR through the U.S. CDC under the terms of NU2GGH001914‐05.

## DISCLAIMER

The findings and conclusions in this report are those of the authors and do not necessarily represent the official position of the funding agencies.

## Data Availability

The data that support the findings of this study are available from the corresponding author upon reasonable request.

## References

[1] Caring for women subjected to violence: a WHO curriculum for training health‐care providers. Geneva: World Health Organization; 2021.

[2] Mecanismo multisectorial de atendimento integrado a mulher vitima de violencia [Multisectoral integrated care mechanism for women victims of violence]. Maputo: Ministry of Health Mozambique; 2012.

[3] Relatorio Annual de Violencia Baseado no Genero [Annual Gender‐Based Violence Report]. Maputo: Ministry of Health Mozambique; 2021.

[4] Cardo DM , Culver DH , Ciesielski CA , Srivastava PU , Marcus R , Abiteboul D , et al. A case control study of HIV seroconversion in health care workers after percutaneous exposure. N Engl J Med. 1997;337:1485–90. 10.1056/nejm. 9366579

[5] Isah A , Igboeli NU , Dim OF , Ekwuofu AA . HIV infections averted at PEPFAR‐APIN clinics in Nigeria: a ten‐year retrospective evaluation of the clinical outcomes of post‐exposure prophylaxis services. Afr J AIDS Res. 2023;22:46–53. 10.2989/16085906. 36951407

[6] Young T , Arens FJ , Kennedy GE , Laurie JW , Rutherford GW . Antiretroviral post‐exposure prophylaxis (PEP) for occupational HIV exposure. Cochrane Database Syst Rev. 2007;2007(1):CD002835. 10.1002/14651858.CD002835.pub. 17253483 PMC8989146

[7] Guidelines on post‐exposure prophylaxis and the use of co‐trimoxazole prophylaxis for HIV‐related infections among adults, adolescents and children: recommendations for a public health approach. Geneva: World Health Organization; 2014. 26042326

[8] Updated recommendations in first‐line and second‐line antiretroviral regimens and post‐exposure prophylaxis and recommendations on early infant diagnosis of HIV. Supplement to the 2016 consolidated guidelines on the use of antiretroviral drugs for treating and preventing HIV infection. Geneva: World Health Organization; 2018.

[9] Guidelines for HIV post‐exposure prophylaxis. Geneva: World Health Organization; 2024. 39259822

[10] Clinical Protocol for Prophylaxis Post Exposure to HIV. Maputo: Ministry of Health Mozambique; 2020.

[11] De Schacht C , Paulo P , Van Rompaey S , Graves E , Prigmore HL , Bravo M , et al. Health care services for survivors of gender‐based violence: a community and clinic‐based intervention in Zambézia Province, Mozambique. AIDS Care. 2023;35(1):16–24. 10.1080/09540121.2022.206731. 35578397 PMC11288795

[12] Kanagasabai U , Valleau C , Cain M , Chevalier MS , Hegle J , Patel P , et al. Understanding gender‐based violence service delivery in CDC‐supported health facilities: 15 sub‐Saharan African countries, 2017–2021. AIDS Educ Prev. 2023;35:39–51. 10.1521/aeap. 37406144

[13] Karim A , Baxter C , Birx D . Prevention of HIV in adolescent girls and young women: key to an AIDS‐free generation. J Acquir Immune Defic Syndr. 2017;75:17–26. 10.1097/QAI.0000000000001316. 28398993

[14] Decker M , Latimore A , Yasutake S , Haviland M , Ahmed S , Blum RW , et al. Gender‐based violence against adolescent and young adult women in low‐ and middle‐income countries. J Adolesc Health. 2015;56(2):188–96. 10.1016/j.jadohealth.2014.09.00. 25620301

[15] Mushambi F , Timire C , Harries AD , Tweya H , Goverwa‐Sibanda TP , Mungofa S , et al. High post‐exposure prophylaxis uptake but low completion rates and HIV testing follow‐up in health workers, Harare, Zimbabwe. J Infect Dev Ctries. 2012;15:559–65. 10.3855/jidc.12214. PMC8655953 33956657

[16] Forda N , Irvinea C , Shubberb Z , Baggaleya R , Beanlanda R , Vitoriaa M , et al. Adherence to HIV postexposure prophylaxis: a systematic review and meta‐analysis. AIDS. 2014;28:2721–7 25493598 10.1097/QAD.0000000000000505

[17] Patton MQ . Qualitative research and evaluation methods. Thousand Oaks, CA: Sage; 2002.

[18] National Action Plan to Respond to Gender‐based Violence in the Health Sector: 2019–2022. Maputo: Ministry of Health Mozambique; 2019.

[19] Braun V , Clarke V . Using thematic analysis in psychology. Qual Res Psychol. 2006;3(2):77–101.

[20] Palermo T , Bleck J , Peterman A . Tip of the iceberg: reporting and gender‐based violence in developing countries. Am J Epidemiol. 2014;179(5):602–12. 24335278 10.1093/aje/kwt295 PMC3927971

[21] Wood S , Glass N , Decker M . An integrative review of safety strategies for women experiencing intimate partner violence in low and middle‐income countries. Trauma Violence Abuse. 2021;22(1)68–82. 30669943 10.1177/1524838018823270

[22] Ellsberg MT , Arango DJ , Morton M , Gennari F , Kiplesund S , Contreras M , et al. Prevention of violence against women and girls: what does the evidence say? Lancet. 2015;385(9977):1555–66. 10.1016/S0140-6736(14)61703-7 25467575

[23] Temmerman M , Ogbe E , Manguro G , Khandwalla I , Thiongo M , Mandaliya KN , et al. The gender‐based violence and recovery centre at Coast Provincial General Hospital, Mombasa, Kenya: an integrated care model for survivors of sexual violence. PLoS Med. 2019;16(8):e1002886. 10.1371/journal.pmed.1002886 31374074 PMC6677296

[24] Osterberg L , Blaschke T . Adherence to medication. N Engl J Med. 2025;353:487e97. 10.1056/NEJMra050100 16079372

[25] Ito H . What should we do to improve patients’ adherence? J Exp Clin Med. 2013;5(4):127e130.

[26] Bentz L , Enel P , Dunais B , Durant J , Poizot‐Martin I , Tourette‐Turgis C , et al. Evaluating counseling outcome on adherence to prophylaxis and follow‐up after sexual HIV‐risk exposure: a randomized controlled trial. AIDS Care. 2010;22:1509–16. 20824548 10.1080/09540121.2010.484457

[27] Roland ME , Neilands TB , Krone MR , Coates TJ , Franses K , Chesney MA , et al. A randomized noninferiority trial of standard versus enhanced risk reduction and adherence counseling for individuals receiving post‐exposure prophylaxis following sexual exposures to HIV. Clin Infect Dis. 2011;53:76–83. 21653307 10.1093/cid/cir333 PMC3110285

[28] National Action Plan to Respond to Gender‐based Violence in the Health Sector: 2024–2030. Maputo: Ministry of Health Mozambique; 2024.

[29] Fulmer T , Feldman P , Kim T , Carty B , Beers M , Molina M , et al. An intervention study to enhance medication compliance in community dwelling elderly individuals. J Gerontol Nurs. 1999;25:6e14. 10.3928/0098-9134-19990801-04 10711101

[30] Hoffman L , Enders J , Luo J , Segal R , Pippins J , Kimberlin C . Impact of an antidepressant management program on medication adherence. Am J Manag Care. 2003;9:70e80. 12549816

[31] Schedlbauer A , Schroeder K , Fahey T . How can adherence to lipid‐lowering medication be improved? A systematic review of randomized controlled trials. Fam Pract. 2007;24:380e7. 17630270 10.1093/fampra/cmm030

[32] Schedlbauer A , Davies P , Fahey T . Interventions to improve adherence to lipid lowering medication. Cochrane Database Syst Rev. 2010;17:CD004371. 10.1002/14651858.CD004371.pub3 20238331

[33] Second Acceleration Response Plan to HIV and AIDS in Mozambique: 2024–2026. Maputo: Ministry of Health Mozambique; 2024.

